# Graph theoretical measures of fast ripple networks improve the accuracy of post-operative seizure outcome prediction

**DOI:** 10.1038/s41598-022-27248-x

**Published:** 2023-01-07

**Authors:** Shennan A. Weiss, Itzhak Fried, Chengyuan Wu, Ashwini Sharan, Daniel Rubinstein, Jerome Engel, Michael R. Sperling, Richard J. Staba

**Affiliations:** 1 Department of Neurology, State University of New York Downstate, Brooklyn, USA; 2 Department of Physiology and Pharmacology, State University of New York Downstate, 450 Clarkson Avenue, MSC 1213, Brooklyn, NY 11203 USA; 3 Department of Neurology, New York City Health + Hospitals/Kings County, Brooklyn, NY USA; 4 Department of Neurology, David Geffen School of Medicine at UCLA, Los Angeles, USA; 5 Department of Neurosurgery, David Geffen School of Medicine at UCLA, Los Angeles, USA; 6 Department of Neurobiology, David Geffen School of Medicine at UCLA, Los Angeles, USA; 7 Department of Psychiatry and Biobehavioral Sciences, David Geffen School of Medicine at UCLA, Los Angeles, USA; 8 David Geffen School of Medicine at UCLA, Brain Research Institute, Los Angeles, CA 90095 USA; 9 Department of Neurology and Neuroscience, Thomas Jefferson University, Philadelphia, USA; 10 Department of Neuroradiology, Thomas Jefferson University, Philadelphia, USA; 11 Department of Neurosurgery, Thomas Jefferson University, Philadelphia, PA 19107 USA

**Keywords:** Neurological disorders, Epilepsy

## Abstract

Fast ripples (FR) are a biomarker of epileptogenic brain, but when larger portions of FR generating regions are resected seizure freedom is not always achieved. To evaluate and improve the diagnostic accuracy of FR resection for predicting seizure freedom we compared the FR resection ratio (RR) with FR network graph theoretical measures. In 23 patients FR were semi-automatically detected and quantified in stereo EEG recordings during sleep. MRI normalization and co-registration localized contacts and relation to resection margins. The number of FR, and graph theoretical measures, which were spatial (i.e., FR rate-distance radius) or temporal correlational (i.e., FR mutual information), were compared with the resection margins and with seizure outcome We found that the FR RR did not correlate with seizure-outcome (*p* > 0.05). In contrast, the FR rate-distance radius resected difference and the FR MI mean characteristic path length RR did correlate with seizure-outcome (*p* < 0.05). Retesting of positive FR RR patients using either FR rate-distance radius resected difference or the FR MI mean characteristic path length RR reduced seizure-free misclassifications from 44 to 22% and 17%, respectively. These results indicate that graph theoretical measures of FR networks can improve the diagnostic accuracy of the resection of FR events for predicting seizure freedom.

## Introduction

One third of patients with focal epilepsy are drug resistant and may benefit from epilepsy surgery. Of the patients who undergo an anterior temporal lobectomy for unilateral mesial temporal lobe epilepsy 70–80% are seizure-free post-operatively^1^, but far fewer patients with neocortical epilepsy achieve seizure freedom after resection^2^. In patients being evaluated in the epilepsy monitoring unit with intracranial electrodes for epilepsy surgery, the location of the seizure-onset zone is used to guide the margins of the resection^3^. Studies in the last two decades have focused on whether this approach can be improved with the inter-ictal identification of fast ripples (FR).

FR are brief (8–50 ms) bursts of spectral energy 200–600 Hz in frequency^4^. In animal models, FR are first generated following insults with toxins, such as kainic acid^5,6^, or after traumatic brain injury^7,8^. The generation of FR by these insults predicts the later development of epileptic seizures on a within subject basis^6–8^. Additionally, FR may be directly involved in ictogenesis. In rodents and humans FR occurs prior to the hypersynchronous^9,10^ and during low-voltage fast onset^10,11^ seizures. Thus, identification and resection of FR alone or resection of both FR and the seizure onset zone (SOZ) could increase the likelihood for seizure free outcome in patients undergoing surgery for medically refractory epilepsy.

In humans FR are rarely recorded from healthy brain tissue^12^. Thus, FR have been investigated as a biomarker of the epileptogenic zone (EZ), which is proposed as necessary and sufficient for seizure generation^3^. To determine if FR define the EZ, measures of the resected and residual FR sites are used to classify a patient’s post-operative seizure outcome. The results from past studies utilizing this approach are difficult to synthesize because of several crucial differences in their implementation including: (1) electrode contact locations and contact type used in each study (subdural^13–15^ vs. Stereo EEG [SEEG]^16–18^); (2) the patient’s level of consciousness during the recording (anesthetized vs. non-REM sleep)^19^; (3) the duration of the iEEG recording used for the analysis^18,20^; (4) the degree of spatial sampling in the analyzed patients’ intracranial electrode implants^18,21^; and (5) the definition of a FR such as frequency criteria^22^, whether the event is superimposed on an epileptiform spike^8,15^, and whether the detection of FR is confounded by recording^15^ or filter ringing artifact^23^.

One recent study examining whether resection of FR predicts seizure freedom using whole night SEEG recordings from extensive implants have shown that if 60% of FR are resected a seizure-free outcome can be predicted in most patients. Despite this, a small proportion of the non-seizure-free patients in this study had resection ratios (RR) that exceeded the 60% FR RR threshold^18^. Another large multi-center study using a similar approach, but shorter recording epochs, observed a similar phenomenon^21^.

In our study, to improve on the FR RR classification accuracy, we developed FR graph theoretical metrics. One metric, the FR rate-distance radius difference, accounts for the spatial distribution of the electrode contacts with FR and thereby estimates differences in spatial sampling. The other metrics are based on FR temporal correlations, which could be as, or more, important than FR rates alone because FR propagate and can organize in networks^24,25^ and patients with no improvement after surgery often exhibit FR networks with more desynchrony^22^. To measure temporal correlations of FR we compared the onset times of FR between contacts using mutual information (MI), which measures non-linear dependencies between time-series or spike trains^26^.

We examined these new FR metrics in a cohort of 23 patients who were implanted with SEEG electrodes and later underwent resection or ablation. We used FR with a higher spectral frequency and found that using the FR graph theoretical measures as a reflex test (*i.e.,* a follow-up test preformed subsequent to initial test results) following the FR RR resulted in a greater number of correct post-operative seizure outcome classifications. Our findings suggest seizure freedom and improvement after surgery may derive from both resection of epileptogenic areas and disconnection of a pathophysiological network in the residual brain tissue^27–32^.

## Results

The study cohort consisted of 23 patients, 12 males and 11 females, and excluded 36 other patients for reasons listed in Fig. 2C. The patients had diverse etiologies of their medically refractory focal epilepsy (Table S1) with 6 of the 23 with normal MRI findings. Overall, the seizures onset zones (SOZ) were localized to the mesial temporal lobe and cingulate cortex in 14 patients, lateral temporal lobe in 7, frontal lobe in 9, and parietal lobe in 3. Five patients had multilobar SOZs. A resection was performed in 21 of these patients with 10 receiving an anterior temporal lobectomy and 2 patients laser ablation performed. In 6 of the 23 patients, the operation was a surgical revision. The post-operative seizure outcome was Engel 1 (E1) for 10 of the patients, E2 for 1 of the patients, E3 for 3 of the patients, and E4 for 9. Because only a single patient had a E2 outcome, the E2 and E3 patients were combined into a single class. The SOZ was completely resected in 3 of 10 of the E1 patients, 2 of 4 of the E2/E3 patients, and 3 of 9 of the E4 patients. Mean time at last follow up was 31.45 ± 3.1 months (standard error of the mean).

**Figure 1:**
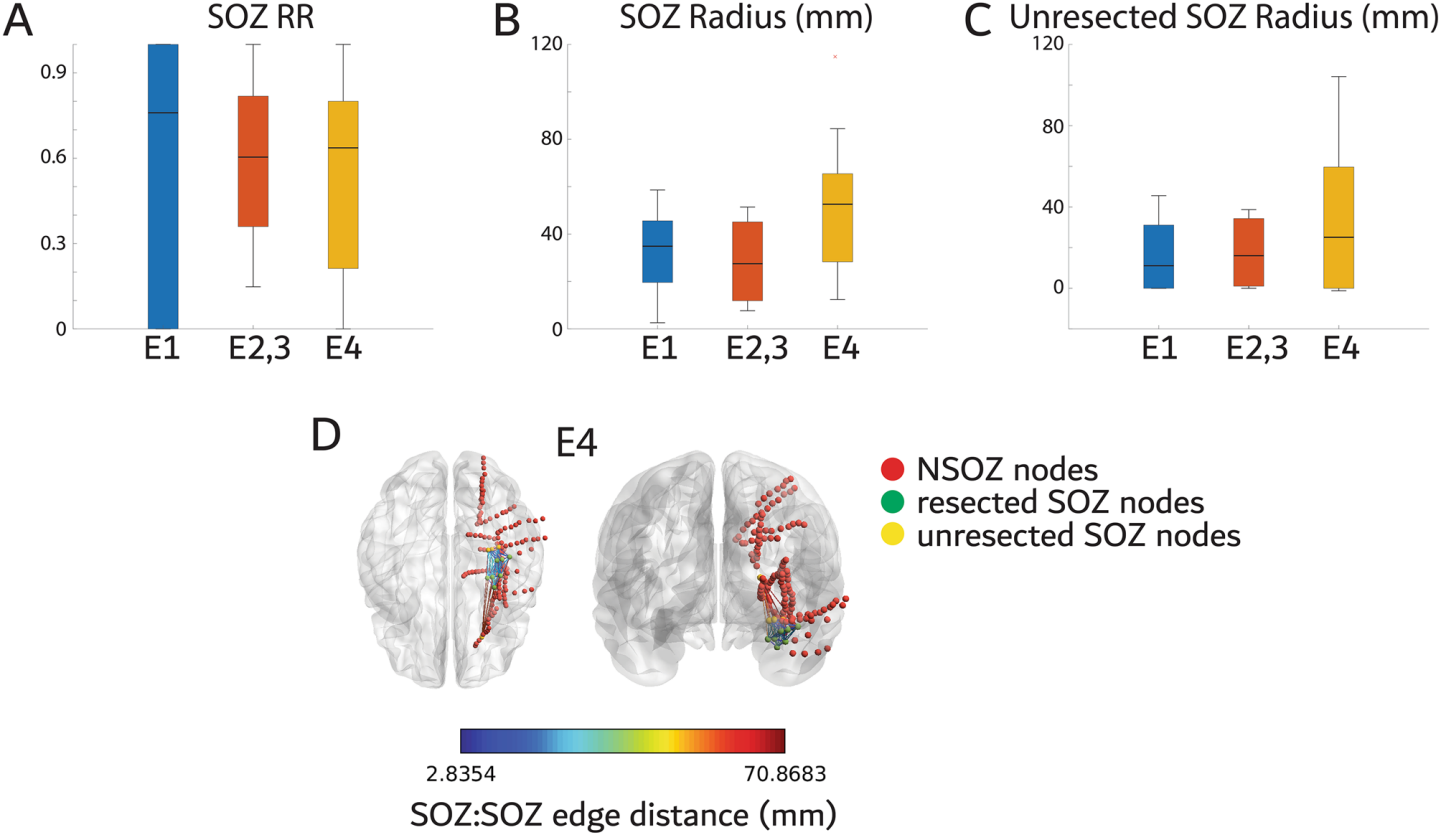
The proportion of the seizure onset zone (SOZ) resected, and the radius of the spatial network of all and unresected SOZ electrodes does not correlate with post-operative seizure outcome. (**A**) Box plot of the SOZ resection ratio (RR) stratified by Engel (E) outcome for all the patients in the cohort (Spearman, *p* > 0.05). (**B**) Box plot of the SOZ radius stratified by Engel outcome (Spearman, *p* > 0.05). (**C**) Box plot of the unresected SOZ radius stratified by Engel outcome (Spearman, *p* > 0.05). (**D**) Illustration of the SOZ distance network for a patient with an E4 outcome. Stereo EEG contacts in the non-SOZ are colored red, the contacts that were in the SOZ and resected are colored green and contacts that were both in the SOZ and unresected are colored yellow. The SOZ to SOZ (SOZ:SOZ) contact edges are color coded by the Euclidian distance (see color bar). The radius is calculated from the network formed by these edges.

We first examined whether the resection ratio (RR) of the SOZ correlated with seizure outcome, and whether the radius of the spatial network formed by the SOZ contacts (*i.e.,* nodes), and the radius of the unresected SOZ network correlated with outcome. We used the Spearman rank correlation test, rather than two sample test, to assess whether each measure monotonically correlates with Engel classes^21^. Results show that the SOZ RR (Fig. 1A, spearman rank correlation, rho = −0.09, *p* = 0.69, r^2^ = 0.01), the SOZ radius (Fig. 1B,D, rho = 0.27, p = 0.21, r^2^ = 0.10), and the unresected SOZ radius (Fig. 1C,D, rho = 0.13, *p* = 0.54, r^2^ = 0.07) did not correlate with outcome. Further details of each patient’s implant and resection with respect to FR generating nodes are provided in Table S2.

### Characterization of the FR > 350 Hz resection ratio (RR) for predicting outcome

**Figure 2:**
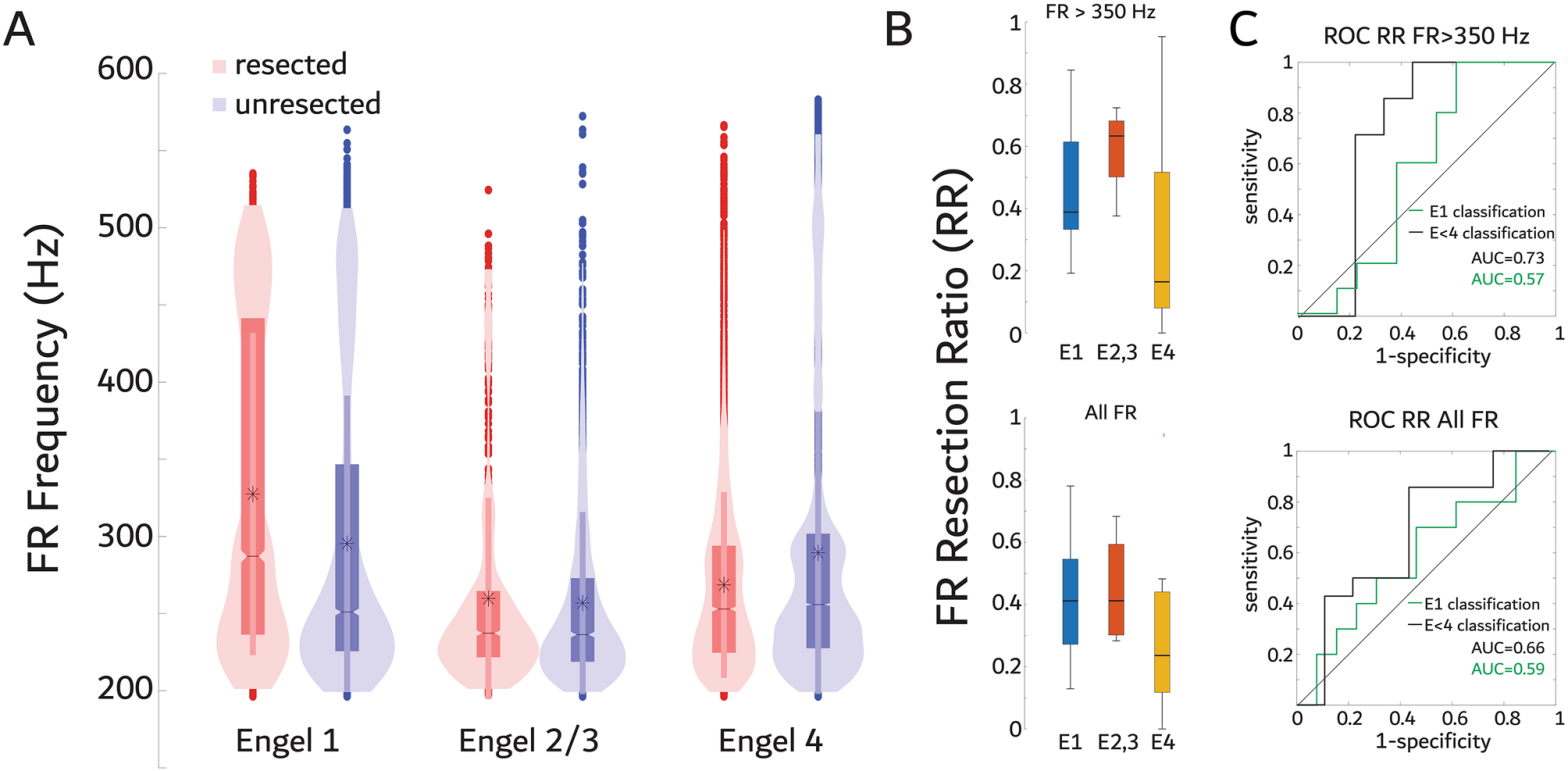
Correlates of fast ripple (FR) resection ratios and relative rates with outcome. (A) Violin plots of FR frequency distribution stratified by the resected electrodes and post-operative seizure outcome Engel (E) class. Fast ripples were increased in frequency in the resection margins of E1 patients (GLMM, *p* < 1e-10), but decreased in frequency in the resection margins of E2-4 patients (GLMM, *p* < 1e-8)(B) Box plot of the FR resection ratio (RR) stratified by Engel class for FR > 350 Hz (top, *p* > 0.05 spearman rank correlation) and all FR irrespective of frequency (bottom, *p* > 0.05 spearman). (C) Receiver operating characteristic (ROC) curves of seizure-free classification (E1,green) and improvement classification (E < 4,black) by FR > 350 Hz rates (top) and all FR rates (bottom) including the area under the ROC curve (AUC). The RR cutoff was 0.192 and 0.366 for FR > 350 Hz and all FR for seizure-free classification, respectively. The RR cutoff was 0.192 and 0.272 for FR > 350 Hz and all FR for improvement classification, respectively.

We first measured the FR RR calculated as the ratio of the total number of resected fast ripples (FR) events divided by total events in each patient to compare with post-operative seizure outcome^16–18,21^. Our prior work had found that FR > 350 Hz were a superior biomarker of epileptogenic brain tissue based on the location of the SOZ^22^. To improve the utility of our FR biomarker measures for predicting outcome we evaluated this hypothesis in our cohort using the resection margins instead. We found that in seizure-free patients (i.e., E1 outcome) electrode contacts in the resection margins had higher-frequency FR on oscillations (FRonO, *p* < 1e-10, Fig. 2A, Table S3), whereas non-seizure free patients had lower-frequency FRonO in the resected contacts (*p* < 1e-8, Fig. 2A, Table S3). We next determined if the FR RR better correlated with outcome and accuracy classified outcome when FRonO > 350 Hz were used in place of all FR irrespective of frequency. For these calculations, fast ripples on spikes (fRonS) were not excluded based on frequency. We found that the FR RR values using either FR > 350 Hz or all FR did not correlate with post-operative outcome (Fig. 2B, spearman rank correlation [FR > 350 Hz, all FR] rho = −0.27, −0.23, *p* = 0.20, 0.30 r^2^ = 0.04, 0.03). Consequently, the area under the receiver operating characteristic (ROC) curve (AUC) for classifying seizure-free patients using the FR > 350 Hz and all FR RRs was only 0.57 and 0.59, respectively (Fig. 2C). In contrast, AUC’s for classification of seizure-improved patients (E1-E3) was 0.73, and 0.66, respectively (Fig. 2C). Overall, at the maximum of Youden’s J of the ROC curve, which is the threshold where sensitivity and specificity are maximized, the accuracy of FR > 350 Hz RR for predicting seizure-free and seizure-improved patients trended slightly better than all FR RR, but still lacked specificity (Table 1). Previous efforts to classify seizure-outcome have examined the complete removal of contacts with a FR rate above a given percentile^19,33,34^. Using this method, we found a small improvement in specificity for classifying seizure-improved patients using FR > 350 Hz, but for classifying seizure-free patients sensitivity improved using all FR irrespective of frequency. FR superimposed on ripples did not achieve any benefit (Fig. S1).

### Characterization of a spatial FR graph theoretical measure for predicting outcome

**Figure 3:**
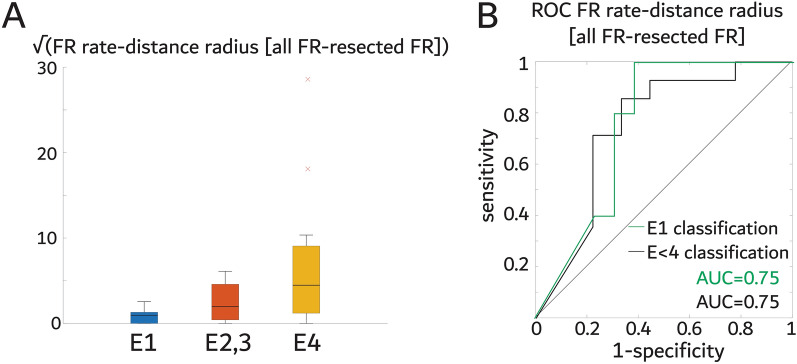
The spatial FR graph theoretical measure the FR rate-distance radius difference between all FR generating contacts minus FR generating contacts in resected regions distinguishes seizure free patients (E1) and seizure improved patients (E1-3). (**A**) Box plot of the square root of the difference of the FR rate-distance radius between all FR generating contacts and only resected FR generating contacts stratified by Engel class (E). The square root is used for visualization purposes only (raw values, spearman rank correlation *p* < 0.05). (**B**) Receiver operating characteristic curve (ROC) of the raw difference of the FR rate-distance radius difference between all the FR generating contacts and the FR generating contacts in the resected regions for classifying seizure free patients, and improved patients.

**Figure 4:**
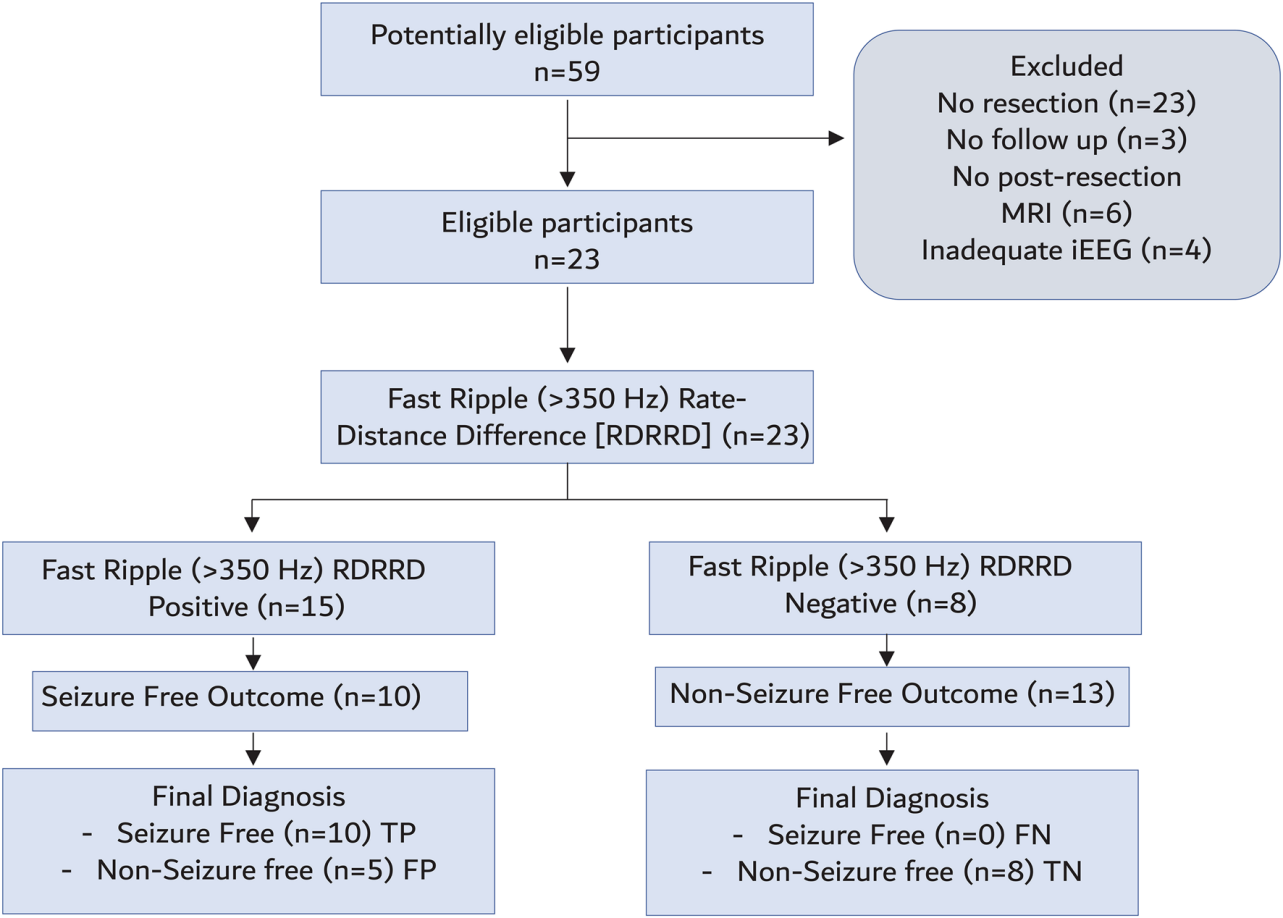
(**A**) STARD flow diagram of the diagnostic accuracy of the FR > 350 Hz rate-distance radius difference measure for predicting seizure freedom. The threshold was determined by the maximum of Youden’s J of the ROC curve (**B**) *TP* true positive, *FP* false positive, *FN* false negative, *TN* true negative.

We next examined whether a graph theoretical measure of a FR spatial network correlated with and classified post-operative seizure outcome. The FR rate-distance radius difference between the whole FR network and the resected network is derived by calculating edge weights between FR generating nodes as the Euclidian distance between the nodes multiplied by the mean FR rate (fRonO > 350 Hz and all fRonS) of each node. The radius of the network is an estimate of the FR generating tissue weighted by rate. The difference between the whole network and the resected network radius is an estimate of the residual FR generating tissue activity. Unlike the FR RR we found that the mean FR rate-distance radius difference did significantly correlate with outcome (Fig. 3A, rho = 0.47, *p* = 0.024, r^2^ = 0.107) and the AUC for classifying seizure-free patients was 0.75 (Fig. 3B). The sensitivity of this measure trended better than the FR > 350 Hz RR (Table 1), but some false positive detections remained (Fig. 4). We also examined the FR distance network radius difference, that was unweighted by rate, and it did not correlate with outcome (Figure S2), indicating that the distance of the nodes in these spatial FR networks was less important as the mean FR rates between the nodes.

### Characterization of temporal correlational FR graph theoretical measures for predicting outcome

**Figure 5:**
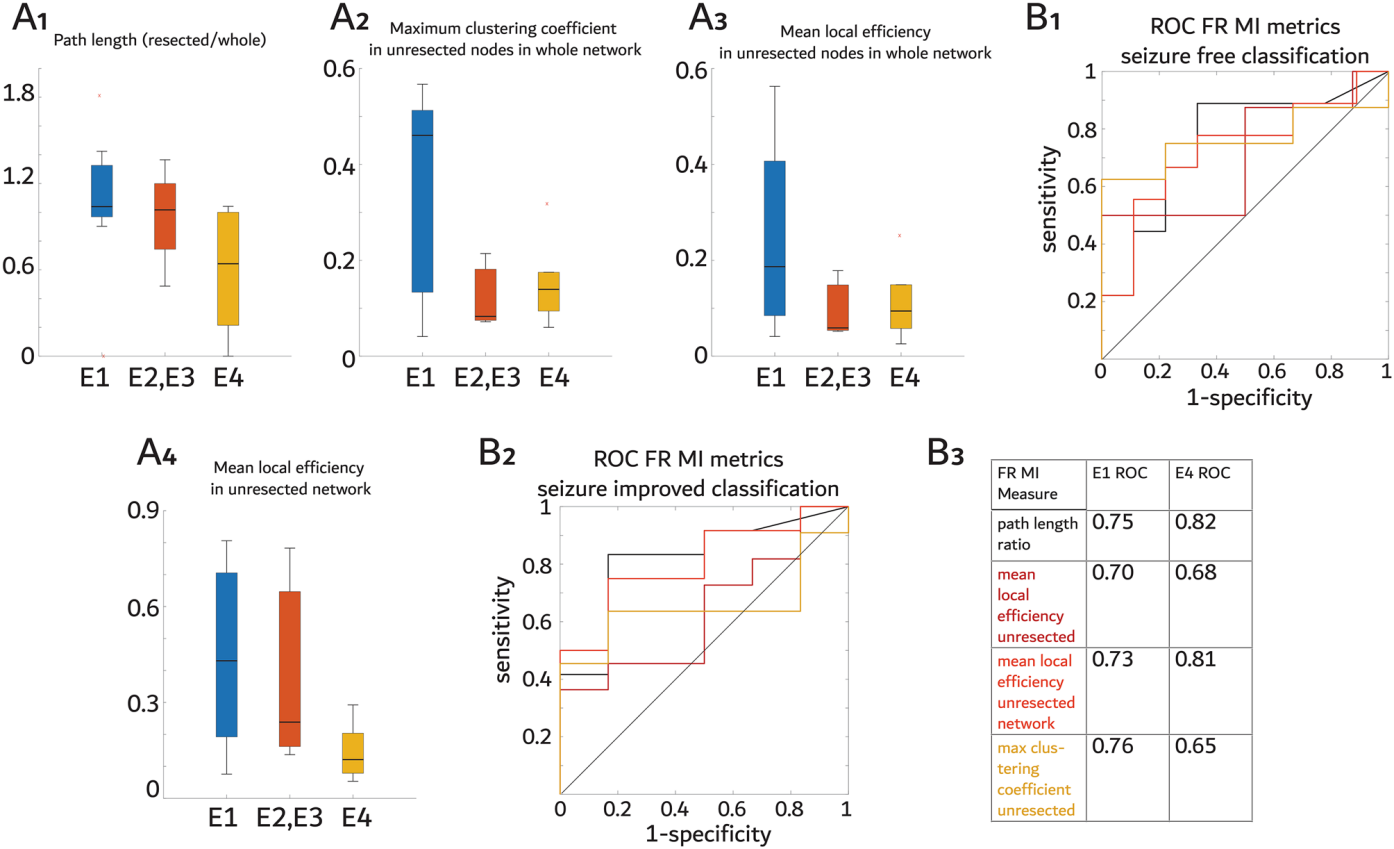
Graph theoretical metrics of the fast ripple (FR) mutual information (MI) temporal correlational network relative to the margins of the resection predict post-operative seizure outcome. (**A**) Box plots of the FR MI graph theoretical metrics stratified by post operative seizure outcome Engel (E) class. The FR MI mean characteristic path length resected ratio (A1) measured from communicating edges only in the resection margins divided by the path length measured from the whole FR MI network (Spearman rank correlation, *p* < 0.05). The maximum clustering coefficient value (A2) among the unresected nodes calculated from the whole FR MI network prior to resection (Spearman, *p* > 0.05). The mean local efficiency value (A3) as in A2 (Spearman, *p* > 0.05). The mean local efficiency value from the FR MI network characterized after resection (Spearman *p* < 0.05, A4). (**B**) Receiver operating characteristic curves (ROC) of these FR MI graph theoretical metrics for predicting seizure free (E1) outcome (B1) or seizure improved (E1-3) outcome (B2). The color-coded legend for the ROC curves and the corresponding area under the ROC curve values (B3).

To explore whether the temporal correlation between FR is associated with post-operative seizure outcome we derived networks with edge weights defined by FR (fRonO > 350 Hz and all fRonS) mutual information (MI). One E1 and one E4 patient had no FR MI network, and one E2 and two E4 patients had the FR MI network completely resected. Of these five patients, three (one E2 and two E4) did not have any nodes with a FR rate greater than three standard deviations greater than all other FR generating nodes. Failure to identify a predominant FR generating node has been associated with poor spatial sampling of epileptogenic regions in prior studies^18^. Thus, three of these five patients may not have had electrode contacts in the EZ. We first examined the mean characteristic path length RR of the FR MI network in the resection margins divided by the whole FR MI network. The mean characteristic path length is the average shortest path between nodes. We found a longer characteristic path length RR correlated with better seizure outcome (Fig. 5A1, rho = −0.512, *p* = 0.03, r^2^ = 0.27), with an AUC for seizure-free classification of 0.75 (Fig. 5B1-3). To examine this result further, we examined the clustering coefficient, the degree to which a node and its neighbors cluster together, and the local efficiency, the inverse of the shortest average path length of a neighborhood around a single node, in the unresected FR MI nodes and within the unresected network. The maximum clustering coefficient of unresected nodes in the FR MI network prior to resection did not correlate with outcome (Fig. 5A2, rho = −0.40, *p* = 0.11, r^2^ = 0.29) and AUC for seizure-free classification was 0.76 (Fig. 5B1-3). The mean local efficiency of unresected nodes in the FR MI network prior to the resection also did not correlate with outcome (Fig. 5A3, rho = −0.35, *p* = 0.17, r^2^ = 0.17) and AUC for seizure-free classification was 0.70 (Fig. 5B1-3). Lastly, a higher mean local efficiency of unresected nodes in the FR MI network constructed from nodes disconnected by the resection correlated with better seizure outcome (Fig. 5A4, rho = −0.47, *p* = 0.047, r^2^ = 0.20) and AUC for seizure free classification was 0.73 (Fig. 5B1-3). These findings suggest that non-seizure-free patients have unresected nodes that have either low FR MI values or are less connected with other neighboring FR nodes.

**Table 1:** Metrics of the confusion matrices derived from each of the fast ripple (FR) resection measures at Youden’s J of the respective receiver operating charecteristic (ROC) curves for predicting post-operative seizure free outcome (top) and improvement in seizures (bottom). Brackets indicate the 95% confidence interval. For the patients without FR mutual information (MI) networks, (i.e., indeterminate) confusion matrices values were imputed appropriately (see supplementary methods).

Sz-Free Prediction	Sensitivity	Specificity	PPV	NPV	Accuracy
FR 350 Hz RR	1 [0.85 1]	0.385 [0.08 0.45]	0.529 [0.45 0.86]	1 [0.85 1]	0.636 [0.55 0.92]
FR all RR	0.7 [0.61 0.95]	0.538 [0.34 0.77]	0.538 [0.2 0.61]	0.7 [0.47 0.87]	0.609 [0.61 0.95]
FR rate-distance	1 [0.85 1]	0.615 [0.31 0.73]	0.667 [0.47 0.87]	1 [0.85 1]	0.783 [0.66 0.97]
FR MI Path length	0.9 [0.52 0.9]	0.462 [0.2 0.61]	0.563 [0.31 0.73]	0.857 [0.66 0.97]	0.652 [0.47 0.87]
FR MI Clust. Coeff	0.667 [0.45 0.86]	0.692 [0.5 0.89]	0.6 [0.45 0.86]	0.75 [0.55 0.92]	0.682 [0.32 0.76]
FR MI Local Eff	0.556 [0.22 0.66]	0.667 [0.34 0.78]	0.556 [0.53 0.92]	0.667 [0.38 0.82]	0.619 [0.43 0.85]
FR MI Local Eff. UR	0.8 [0.52 0.9]	0.462 [0.31 0.73]	0.533 [0.34 0.77]	0.75 [0.66 0.97]	0.609 [0.43 0.84]

Responder Prediction	Sensitivity	Specificity	PPV	NPV	Accuracy
FR 350 Hz RR	1 [0.85 1]	0.556 [0.43 0.84]	0.778 [0.52 0.9]	1 [0.85 1]	0.826 [0.61 0.95]
FR all RR	0.857 [0.61 0.95]	0.556 [0.47 0.87]	0.75 [0.72 0.99]	0.714 [0.52 0.9]	0.739 [0.52 0.9]
FR rate-distance	0.857 [0.66 0.97]	0.667 [0.52 0.90]	0.80 [0.61 0.95]	0.75 [0.39 0.80]	0.783 [0.52 0.90]
FR MI Path length	0.833 [0.61 0.95]	0.636 [0.34 0.77]	0.714 [0.39 0.8]	0.778 [0.47 0.87]	0.739 [0.56 0.93]
FR MI Clust. Coeff	0.636 [0.45 0.86]	0.636 [0.5 0.89]	0.636 [0.45 0.86]	0.636 [0.28 0.72]	0.636 [0.45 0.86]
FR MI Local Eff	0.4 [0.22 0.66]	0.727 [0.48 0.89]	0.571 [0.3 0.74]	0.571 [0.43 0.85]	0.571 [0.43 0.85]
FR MI Local Eff. UR	0.75 [0.56 0.93]	0.636 [0.31 0.73]	0.692 [0.47 0.87]	0.7 [0.47 0.87]	0.696 [0.52 0.9]

**Figure 6:**
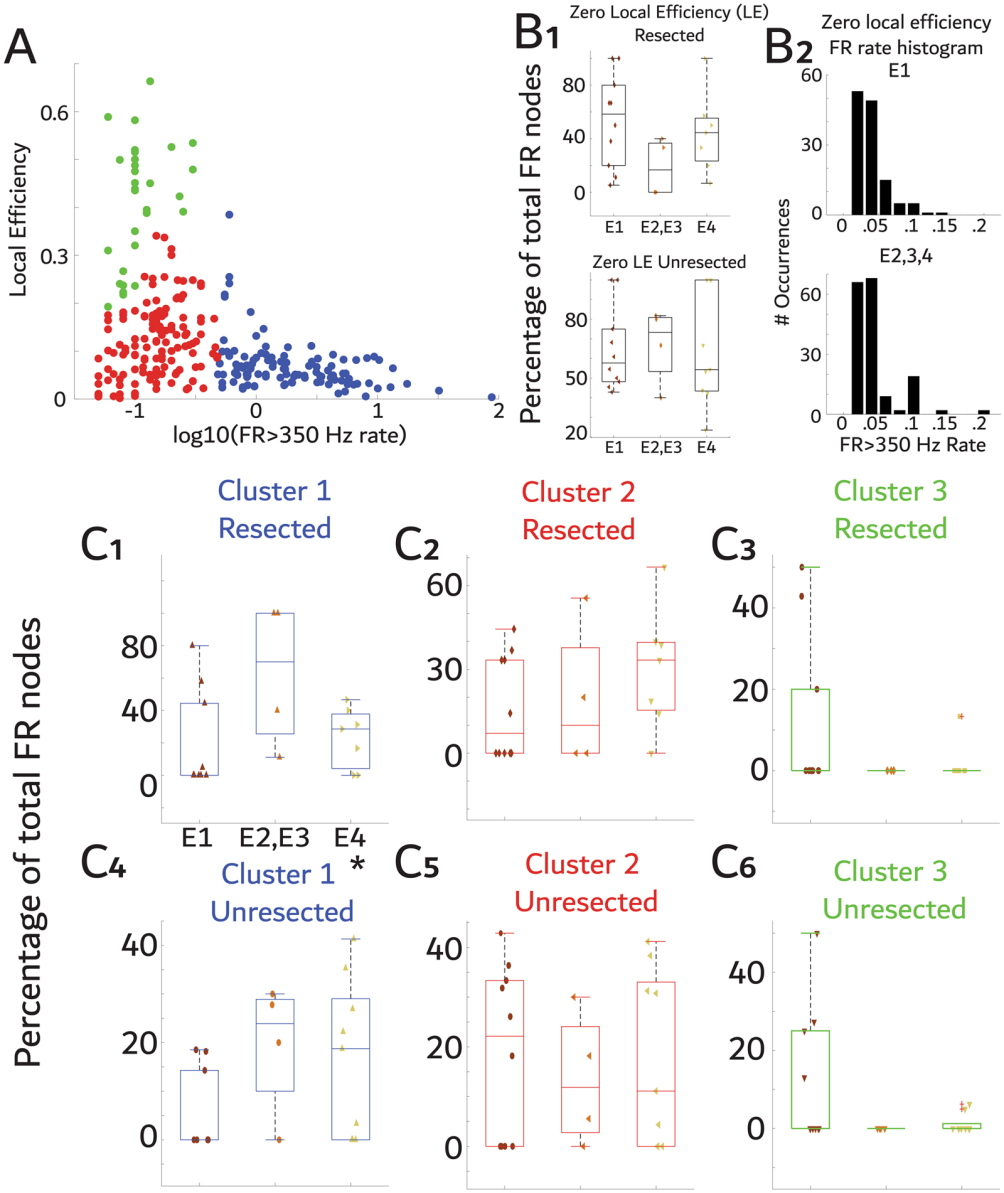
Failure to resect nodes with high FR rates and low local efficiency (*i.e.*, FR desynchrony) correlates with poor surgical outcome. (**A**) Comparison of local efficiency (LE) with FR rate. K-means clustering identified three distinct clusters of nodes. (**B1**) Percentage of resected (top) and unresected (bottom) nodes, within patients, with FR but zero LE (i.e., disconnected and not shown in panel A) for all the patients in the study cohort stratified by outcome. The percentage of zero LE nodes resected or unresected did not correlate with outcome (spearman, *p* > 0.05). (**B3**) Histogram of FR rate in the nodes with zero local efficiency stratified by seizure freedom showing no marked differences. (**C1-3)** The percentage of nodes, within patients, in the resection cavity belonging to each cluster shown in panel A: cluster 1 members (blue, C1); or cluster 2 members (red, **C2**); or cluster 3 members (green, **C3**) stratified by outcome. (**C4-C6**) as in C1-C3 but for unresected nodes. Only cluster 1 unresected nodal percentage was statistically correlated with outcome (C4, asterisk, Spearman, *p* < 0.05). Notably, some of the E1-3 patients also showed relatively higher cluster 1 values in resected nodes (**C1**).

**Figure 7:**
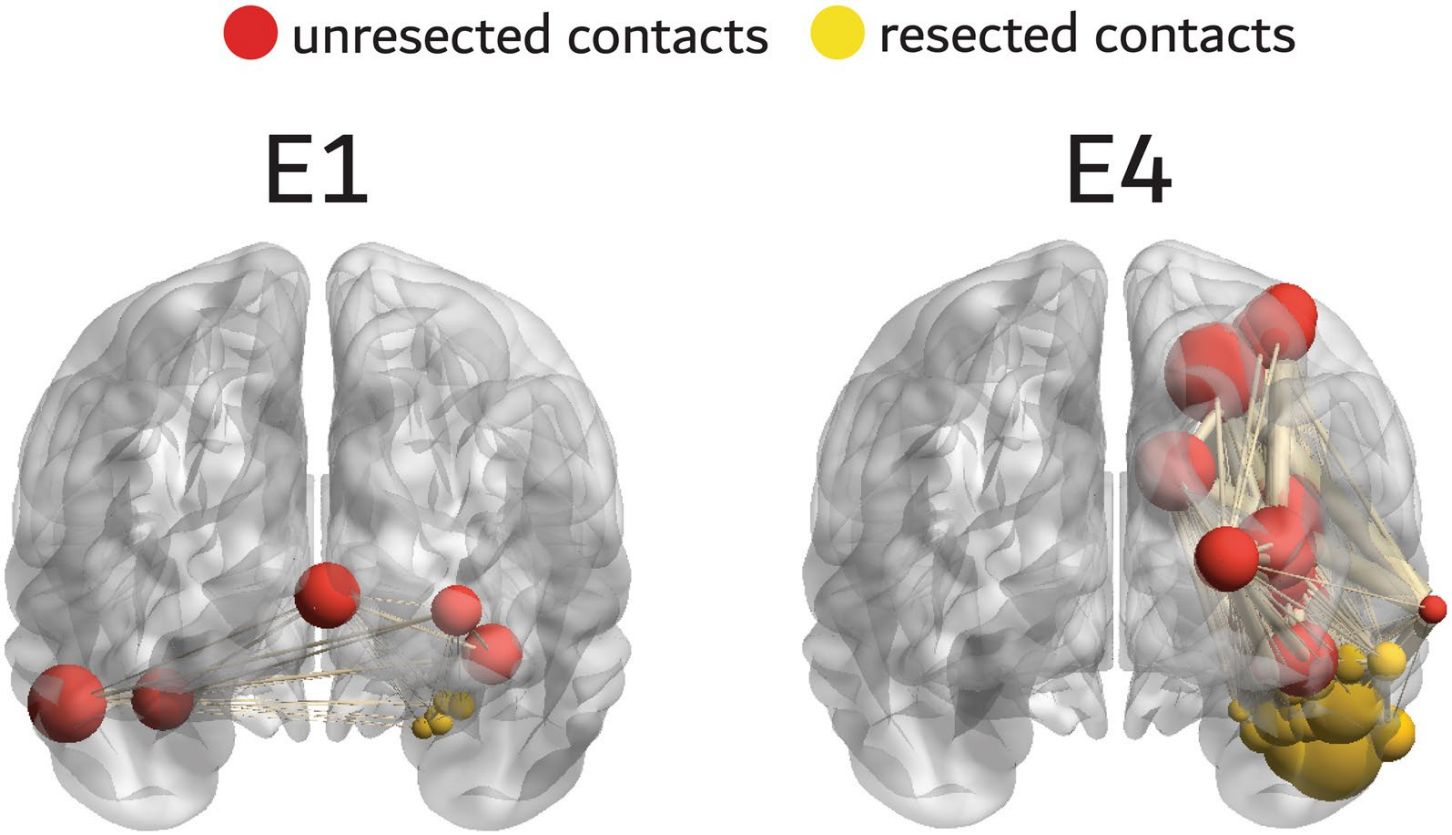
Illustration of the fast ripple (FR) mutual information networks of two patients with Engel 1 (E1, left) and E4 (right) outcome. The resected nodes are colored yellow, and unresected nodes red. The size of the node corresponds to the node’s local efficiency (LE). The edge size corresponds to the FR MI value. In the patient with Engel 1 (E1) outcome, resected contacts exhibit lower fast ripple (FR) mutual information (MI) network LE, and unresected contacts a higher FR MI network LE. In the patient with E4 outcome the resected nodes have higher LE and several unresected nodes have relatively lower LE.

To better understand the FR graph theoretical measures, we compared local efficiency in each node with the FR > 350 Hz rate at that node. We found that nodes with highest FR rate tended to have lower local efficiencies (Fig. 6A). After clustering the nodes (Fig. 6A) we found that a worse outcome was associated with a higher proportion of unresected high-rate and low local efficiency FR generating nodes (Fig. 6C4, rho = 0.435, *p* = 0.04, r^2^ = 0.15). We did not observe significant correlations for the other clusters in the resected (Fig. 6C1-3) or unresected regions (Fig. 6C5-6) or for nodes with zero local efficiency (*p* > 0.05, Fig. 6B1,2). A braingraph illustration of FR MI networks and nodal local efficiency in two patients, one with a E1 outcome and one with a E4 outcome, demonstrates that the E1 patient had low local efficiency FR nodes in the resection margins and relatively high local efficiency FR that were unresected whereas the opposite was true in the E4 patient. In the latter case, at least several relatively low FR local efficiency nodes remained unresected (Fig. 7).

Since the FR MI graph theoretical measure results may have been driven by the number of nodes in the FR MI networks, as opposed to the values of FR MI, we performed a control experiment in which the graph theoretical measures were derived on unweighted networks consisting of the same nodes and edges identified in the FR MI networks. We found that these FR graph theoretical measures performed near chance for classifying seizure free patients (Figure S3). This indicates that the weights of the graphs which are defined by the FR MI are required for seizure outcome classification and that seizure free patients do not simply have fewer edges with MI > 0.

### Reflex testing using FR Graph theoretical measures

**Figure 8:**
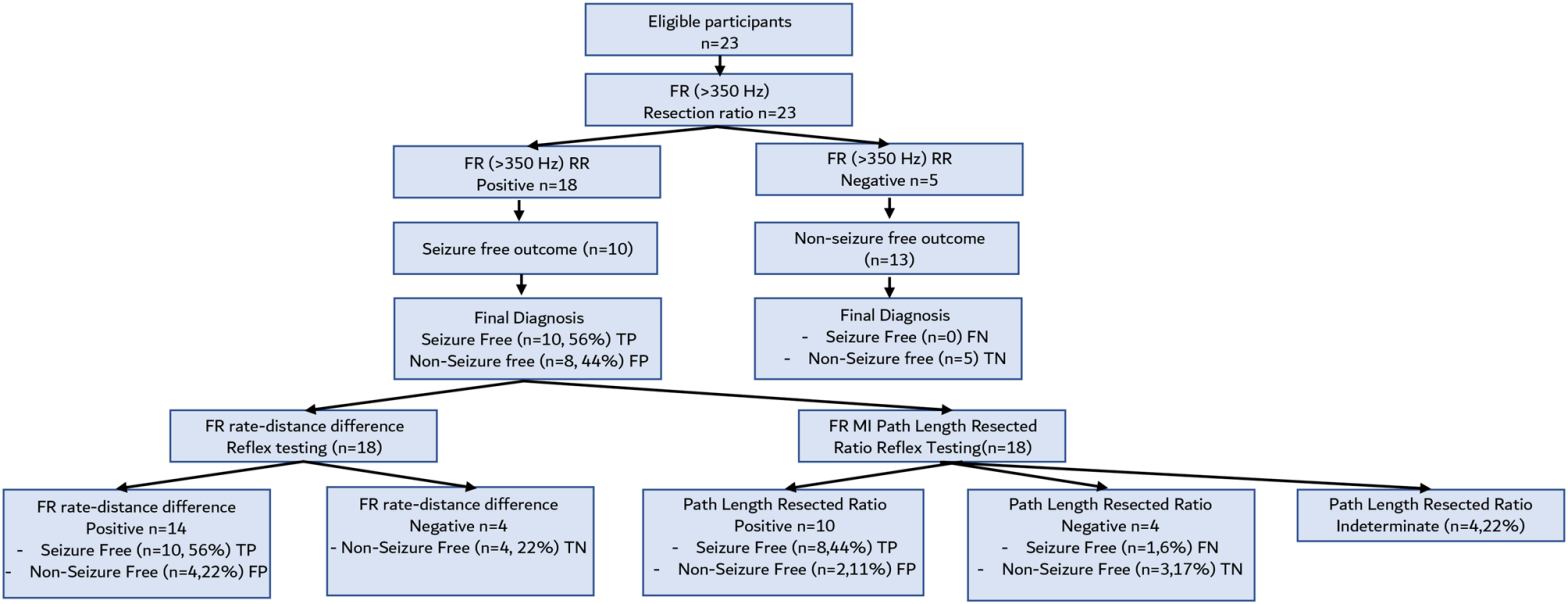
Flow chart of diagnostic accuracy of the fast ripple (FR) > 350 Hz resection ratio (RR) for diagnosing post-operative seizure freedom with reflex testing using either the FR rate-distance difference or the FR mutual information (MI) characteristic path length resected ratio. The thresholds for the tests were determined by the maximum of Youden’s J of the respective receiver operating characteristic curves (not shown). *TP* true positive, *FP* false positive, *FN* false negative, *TN* true negative.

Due to the limited size of our study, we could not demonstrate that any of the FR graph theoretical measures exhibited a superior accuracy to classify seizure outcome than measures using FR > 350 Hz RR. Instead, since the FR RR exhibited low specificity for classifying seizure freedom we asked if the FR rate-distance difference and the mean characteristic path length resected ratio could be used as an effective reflex test for FR RR > 350 Hz positive patients. Reflex testing using either FR > 350 Hz rate-distance radius or the FR > 350 Hz MI mean characteristic path length resected ratio reduced seizure-free misclassifications from 44 to 22% and 17%, respectively (Fig. 8). We also examined reclassification of the FR MI false positives using all the graph theoretical measures in the individual patients and found that each measure correctly reclassified most of the false positives (Table S4).

### Resection of FR propagation sites and outcome

**Figure 9:**
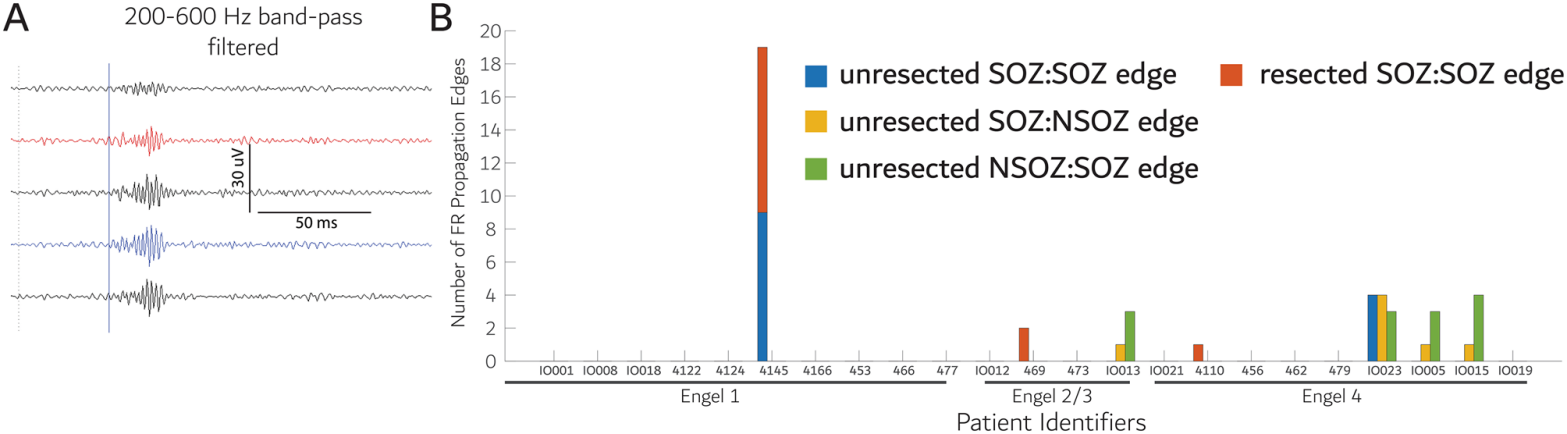
Resection of the electrode contacts where fast ripples (FR) on oscillations propagate from does not reliably correlate with post-operative seizure outcome. (**A**) an example of a fast ripple on oscillation propagation event from the intracranial EEG (iEEG) recorded from the contact in the blue trace to that from the contact in the red trace. All FR in the contact in red reliably followed those FR from the contact in blue (sign test, *p* < 0.005). (**B**) Stacked grouped bar graph of the correlation of outcome in individual patients with the number of contact pairs (i.e., edges) exhibiting FR propagation that were resected or left intact. Only a small minority of the patients exhibited FR propagation and complete resection of FR propagation sites did not correlate with seizure free outcome in any of the patients.

Lastly, we explored whether resecting all sites with propagating inter-ictal fRonO^25^ is associated with seizure freedom. In our patient cohort, we derived the contact pairs (i.e. edges) showing statistically significant FR propagation in and bordering the SOZ (Fig. 9A, sign test, *p* < 0.005). Qualitatively, we found that only ~ 30% of the patients exhibited at least a single edge with FR propagation. One seizure-free patient had a partial (~ 60%) resection of the out-nodes of the FR propagation edges, but among the non-seizure-free patients two had complete resection of the out-nodes of the FR propagation edge(s) and four patients did not (Fig. 9B). ). In this analysis, very few patients had propagating edges and in most patients who did, the number of propagating edges was relatively small. Thus, while complete resection of the origin of FR propagation seemingly does not accurately predict post-operative seizure outcome the results are inconclusive.

## Discussion

Resection of the SOZ is the gold standard for planning a resective/ablative epilepsy surgery. We found that neither the SOZ RR or spatial measures of the full or residual SOZ, that is left unresected, correlated with post-operative seizure outcome, which is in accord with other past observations^16,35–38^. We also found that the FR RR did not significantly correlate with outcome, irrespective of whether FR > 350 Hz or all FR were used in the measure, but FR > 350 Hz showed trends of a modestly more accurate seizure-improved classification compared with all FR. The FR > 350 Hz RR did classify patients at high sensitivity, but low specificity and a lower than ideal RR cutoff value. In contrast to the FR RR, the graph theoretical measure FR > 350 Hz rate-distance radius resected difference did significantly correlate with post-operative seizure outcome and classified seizure-free patients with an accuracy that trended better than the FR > 350 Hz RR. The FR > 350 Hz MI network measure characteristic path length RR also significantly correlated with post-operative seizure outcome and exhibited non-inferior classification accuracy to the FR > 350 Hz RR. However, FR MI network could not be derived in 5 of the 23 patients likely due to poor spatial sampling of the EZ by the SEEG electrodes. Reflex testing of FR > 350 Hz RR positive patients using the FR > 350 Hz rate-distance difference and the FR > 350 Hz MI characteristic path length resected ratio reduced the number of misclassifications, demonstrating the diagnostic utility of these measures in this patient cohort.

The ratio of FR that need be resected to achieve a seizure-free outcome has been a matter of debate. Using brief 5–10-min intraoperative recordings from subdural electrodes it has been shown that a single unresected FR can predict a non-seizure-free outcome^14,39^. However, longer duration recordings from SEEG electrode contacts during sleep contradict this and have derived a FR RR cutoff as high as 0.6 for classification of post-operative seizure outcome^16–18,21^. In our cohort many of the seizure-free patients had RR values lower than 0.6. Thus, in our study the RR cutoff used was 0.192 for FR > 350 Hz and 0.366 for all FR. Previous studies have found that the FR RR optimally classifies patients with mesial-temporal lobe epilepsy and in our cohort only 10 patients had anterior temporal lobectomies^17^. Furthermore, our cohort included 6 patients with repeat operations. It is possible that these demographics contributed to the low FR RR values calculated in this study’s seizure-free patients. In accord with this hypothesis, multicenter studies have shown that the utility of the FR RR approach for classifying outcome may be dataset dependent^21^. Our selection of FR > 350 Hz, which appears to have influenced the low FR RR cut-off, was based on the statistically significant GLMM model results (Table S3) and visual inspection of Fig. 2A as well as consistency with our prior work^22^. FR > 350 Hz RR trended more accurate in predicting seizure-improved outcome than all FR, suggesting a larger study is needed. Notably, very fast and ultrafast ripples with spectral frequency > 500 Hz have been reported as specific biomarkers of the EZ, previously^40–42^. Overall, our RR results suggest that only a small ratio of FR > 350 Hz need to be resected to achieve seizure freedom or improvement but more than half of the patients that were non-seizure free had FR > 350 Hz RR exceeding the cutoff (Fig. 4).

The FR rate-distance difference metric was significantly correlated with outcome and the accuracy of outcome classification trended better than the FR > 350 Hz RR. The FR rate-distance difference also reduced false positive detections when used as a reflex test after the FR > 350 Hz RR test. To conceptualize the difference between the FR RR and the FR rate-distance difference consider one SEEG electrodes implanted in the hippocampus, one SEEG electrode implanted in the ipsilateral orbitofrontal cortex and one SEEG electrode implanted in the ipsilateral parietal lobe. Assuming a very high rate of FR in the hippocampus and moderate rates in the orbitofrontal cortex and parietal lobe, if the patient undergoes an anterior temporal lobectomy, then the RR will be high (i.e., predicting seizure freedom). However, the rate-distance difference metric also will be high (i.e., predicting non-seizure freedom) because of the distance between the orbitofrontal and parietal SEEG electrodes that are detecting FR. The FR rate-distance difference may account for spatial under-sampling of FR generating sites between the SEEG electrodes.

The FR MI network characteristic path length RR and mean local efficiency within the unresected network correlated with post-operative seizure outcome. Furthermore, reflex testing of the FR > 350 Hz RR positives using the mean characteristic path length RR reduced misclassifications. The neurophysiological substrates of the FR temporal correlations measured by MI are unclear. FR have been found to propagate within the SOZ^24,43^, and sometimes precede epileptiform discharges^25^. One study suggested that resecting sites of propagation could be important for predicting seizure-freedom. Our results like the ones from latter study derive from a small sample and are inconclusive, but our data suggest the statistical criteria and assumptions of the sign test, which is used to establish FR propagation, are too strict, and unlikely to reflect physiological network topologies, since FR propagation cannot be demonstrated in a single instance (*i.e.,* edge) in most patients. Potentially, a diverse number of physiological mechanisms are captured by the MI measure, which in some cases may not relate to FR propagation but could relate to FR synchrony^44^. Using k-means clustering analysis we found that nodes with the highest FR rates also had the lowest local efficiencies which implies low MI values, or reduced connectivity, in the edges of this node and its neighbors. Resection of the nodes in this high FR rate and low local efficiency cluster correlated with seizure outcome on a within subject basis. This, alongside with our primary FR MI graph theoretical measure findings, argues that a non-seizure free outcome will occur if brain regions that generate high rates of FR, which are temporally uncorrelated with neighboring sites, are left intact. These results also imply that in planning an effective surgery, brain regions that generate synchronized FR at lower rates may be non-essential. Further examination of this issue is critical since it appears resecting all FR is not required for a seizure free outcome^16–18,21^.

## Limitations

This study was relatively small^18,21^, and as noted the cohort was diverse and may not represent patients treated at most tertiary epilepsy center. Our results demonstrate that the study was not adequately powered to detect differences in accuracy between FR graph theoretical measures and FR RR and perform multiple comparison testing. To overcome these limitations, we compared the results of Spearman rank correlation testing, and implemented reflex testing of the FR > 350 Hz RR positives using our best performing graph theoretical measures and demonstrated a substantial reduction in misclassifications. A larger study is needed to determine if the FR graph theoretical measures are superior to the FR RR, and further explore the utility of the reflex testing approach. One paradoxical finding was the complete resection of the FR MI network in two patients not seizure free. While this could be attributed to spatial under-sampling, other explanations are possible. In general, SEEG studies are limited by the location of the electrode contacts and in some cases, the hypotheses guiding the placement of electrodes do not completely sample epileptogenic regions, which will decrease the accuracy of FR-based measurements for classifying seizure outcome. In fact, we found evidence for spatial undersampling in 3 of the 5 patients who had incomplete FR MI networks who were not seizure free after surgery. Also, our study did not examine ictal network dynamics which have been shown to be valuable in planning an efficacious resection^29–31^. Future work could utilize complementary approaches examining both inter-ictal FR network dynamics and ictal dynamics.

## Conclusion

In summary, we found graph theoretical measures of FR, but not the FR RR, correlates with post-operative seizure outcome. Reflex testing of FR (> 350 HZ) RR positive patients using two of these measures reduced the number of outcome misclassifications. Larger cohort studies are needed, though our results suggest outcome classification could be optimized by combining FR-based metrics using decision tree or other types of machine learning. Such an approach would also allow prediction on the individual patient level in a test set without predetermined thresholds. Also, results are consistent with the epileptic network hypothesis of seizure generation^28,32,45^, and suggest that effective resections can be better planned through examination of temporal correlations of FR.

## Methods

### Patients

This retrospective study of diagnostic accuracy used consecutive recordings selected from 8 patients who underwent intracranial monitoring with depth electrodes between 2014 and 2018 at the University of California Los Angeles (UCLA) and from 15 patients at the Thomas Jefferson University (TJU) in 2016–2018 for the purpose of localization of the SOZ. All methods were carried out in accordance with relevant guidelines and regulations. Data collection was planned before conceptualization of the graph theoretical measures. Inclusion criteria for this study included pre-surgical MRI for MRI-guided stereotactic electrode implantation, as well as a post-implant CT scan to localize the electrodes, and stereo EEG recordings during non-rapid eye movement (REM) sleep at a 2 kHz sampling rate. Patients were excluded if: (1) no resection/ablation was and performed; (2) a post-resection/ablation MRI was not obtained; (3) no adequate post-operative clinical follow up; and (4) A failure to record at least ten minutes of artifact free iEEG during non-REM sleep. Verbal and written informed consent was obtained from all patients and/or their legal guardian(s) prior to participating in this research, which was approved by the UCLA and TJU institutional review boards. Eligible patients were identified through queries of pre-existing clinical databases. The epileptologist defined SOZ was aggregated across all these seizures during the entire iEEG evaluation for each patient^22^.

### Neuroimaging

T1- pre-implant and post-resection MRIs were obtained for each patient. Post-implantation CT scans were then co-registered and normalized with the MRIs using Advanced Neuroimaging Tools (ANTs)^46^ with neuroradiologist supervision, using an in-house pipeline (https://github.com/pennmem/neurorad_pipeline). The position of each electrode contact was localized to normalized MNI coordinates and the Desikan-Killiany atlas^47^. Identification of the named electrode contacts in the resection cavity was performed manually in itk-SNAP.

### EEG recordings and HFO detection

For each patient, clinical iEEG (0.1–600 Hz; 2000 samples per second) was recorded from 8 to 16 depth electrodes, each with 7–15 contacts, using a Nihon-Kohden 256-channel JE-120 long-term monitoring system (Nihon-Kohden America, Foothill Ranch, CA, USA). A larger number of electrodes with more contacts were implanted at TJU. The reference signal used for the recordings performed at UCLA was a scalp electrode position at Fz in the International 10–20 System. The reference signal used for the TJU recordings was an electrode in the white matter that was: (1) estimated far from the cortical layers based on the electrode design and trajectory; (2) far from the putative SOZ based on scalp recordings; and 3) exhibited low voltages relative to the other contacts on the electrode. HFOs and sharp-spikes were detected in the non-REM sleep iEEG using previously published methods (https://github.com/shenweiss) ^15,19,48–50^ implemented in Matlab (Mathworks, Natick, MA, USA) (Supplementary Methods) from 10 to 60 min, per patient. HFOs were detected in both the bipolar and referential montage (Supplementary Methods) and in each case, for UCLA and TJU patients, detection sensitivity and accuracy was typically 80–90%^50^. The different HFO types: (1) ripples on oscillations (RonO); (2) ripples on spikes (RonS); (3) fast ripples on oscillations (fRonO); (4) fast ripples on spikes (fRonS) were distinguished using the topographical analysis of the wavelet convolution^48^. In brief this method distinguished HFOs on spikes by comparing onset of the outermost closed-loop contour of the HFO with onset of the outermost open-loop contour of the spike and determining if the latter occurred earlier. Ripples and fast ripples were distinguished by the mean frequency of the closed-loop contour group^48^. Following automatic detection of HFO and sharp-spikes, false detections of clear muscle and mechanical artifact were deleted by visual review in Micromed Brainquick (Venice, Italy).

### SOZ resection ratio, SOZ radius, and unresected SOZ radius

The SOZ resection ratio was calculated as the proportion of electrode contacts that were clinically labeled as SOZ that were within the resection margins as the numerator and the total number of the SOZ contacts in the denominator. The SOZ and resected SOZ radius were calculated using an adjacency matrix of the Euclidian distance between the all the contacts in normalized MNI coordinates and assigning contact pairs outside the SOZ, or outside both the SOZ and the resection margins to infinite values (Supplementary Methods). The unresected SOZ radius was then calculated as the SOZ radius minus the resected SOZ radius. Graphs were visualized using Brainnet Viewer^51^.

### Fast ripple derived predictors of post-operative seizure outcome

We identified the patients with potential spatial undersampling by calculating the normalized FR rate across all contacts generating at least one fast ripple, within patients, and ascertaining if no contacts exceeded three standard deviations above the mean. The FR RR was derived as the number of FR events on removed channels divided by total number of FR events on all channels. Percentiles of FR rates across contacts within patients were calculated using the prctile.m function in Matlab. All graph theoretical measures were calculated using the Brain Connectivity Toolbox (https://sites.google.com/site/bctnet/)^52^ (Supplementary Methods). The adjacency matrix for the FR rate–distance networks was calculated by the average rate (/min) of the events recorded by two respective nodes multiplied by the Euclidian distance (mm) between these nodes. The adjacency matrix for the mutual information (MI) networks were calculated using FR event ‘spike trains’ defined by the onset times of each event and then calculating MI between nodes with the adaptive partition using inter-spike intervals MI estimator^26^. Using these adjacency matrices, and their inverses, the radius of the FR rate-distance network, mean characteristic path length, clustering coefficient, and local efficiency of the FR MI network were derived, as well as their related values (Supplementary Methods).

### Fast ripple propagation

To identify the contact pairs with statistically significant FR on oscillation (FRonO) propagation, for every pair of channels, we identified the FR starting < 250 ms apart and calculated the delay between the onset times of these events using the meshgrid.m function in Matlab. We then tested whether the median delay between these events was significantly different from zero (non-parametric sign test, *p* < 0.005, Supplementary Methods). The leading contact of the propagation was then compared with the resection margins and the post-operative seizure outcome.

### Statistics

Prior to, and during, statistical analyses the post-operative seizure outcome was unblinded. True positives were defined as patients who were seizure-free (Engel 1) or seizure-improved (Engel 1–3) and had a larger FR resection ratio, lower FR rate-distance radius difference, and larger value of a FR MI graph theoretical measure. Receiver operating characteristic (ROC) curves were generated using the perfcurve.m function in Matlab. Youden’s J was calculated as the sensitivity + specificity-1 of the ROC curve. The confusion matrix was calculated at Youden’s J for post-operatize seizure outcome classification and was used to derive the sensitivity, specificity, positive predictive value (PPV), negative predictive value (NPV), and accuracy. Violin plots and box plots were generated in Matlab. Estimates of the 95% CIs of these measures used the binomial method. Statistical comparisons of the measures across the Engel 1, Engel 2 and 3 (2–3), and Engel 4 patient groups were performed in Matlab using Spearman rank correlation (spearman.m). Additionally, the fRonO frequency was fit with generalized linear mixed-effects models (GLMMs) in Matlab (fitglme.m) with patient as the random-effects term, and resection and neuroanatomical location as fixed-effects predictors.

## Data availability

iEEG recordings are available upon reasonable request from Dr. Shennan Weiss. The iEEG data used for figure generation are available at https://www.zenodo.org/record/6529724#.Ynkv4YfML9Y and https://zenodo.org/record/6532325#.YnlLF4fML9Y. HFO and electrode contact MongoDB JSON files are available at https://zenodo.org/record/6451900#.YmgQie3ML9Y and open-source code for statistics and figure generation from https://github.com/shenweiss/publishedcode.

## Supplementary Information

Supplementary Information.

## Abbreviations

HFO: High-frequency oscillation
FR: Fast ripple
SEEG: Stereo EEG
iEEG: Intracranial EEG
SOZ: Seizure-onset zone
REM: Rapid eye movement
EZ: Epileptogenic zone
RR: Resection ratio
E: Engel
fRonO: Fast ripple on oscillation
fRonS: Fast ripple on spike
ROC: Receiver operating characteristic
AUC: Area under the receiver operating characteristic curve
