# Supplementary material for: Graph theoretical measures of fast ripple networks improve the accuracy of post-operative seizure outcome prediction
Source: Sci Rep. 2023 Jan 7;13:367. doi: 10.1038/s41598-022-27248-x (PMC9825369; doi:10.1038/s41598-022-27248-x)
Supplement: Supplementary file 1 — Supplementary Information. [file 41598_2022_27248_MOESM1_ESM.pdf]

Supplementary Materials

Graph theoretical measures of fast ripple networks improve the accuracy of post-operative seizure outcome prediction

Shennan A Weiss[1,2,3], Itzhak Fried[5], Chengyuan Wu[10,11]
Ashwini Sharan[11], Daniel Rubinstein[9], Jerome Engel Jr[4,5,6,7,8], Michael R. Sperling[9], Richard J Staba[5][4].

[1]Dept. of Neurology, [2]Dept. of Physiology and Pharmacology, State University of New York Downstate, Brooklyn, New York, 11203 USA
[3]Dept. of Neurology, New York City Health + Hospitals/Kings County, Brooklyn, NY, USA
[4]Dept. of Neurology, [5]Dept. of Neurosurgery, [6]Dept. of Neurobiology, [7]Dept. of Psychiatry and Biobehavioral Sciences, [8]Brain Research Institute, David Geffen School of Medicine at UCLA, Los Angeles, California, 90095, USA
[9]Depts. of Neurology and Neuroscience, [10]Dept. of Neuroradiology, [11]Dept. of Neurosurgery, Thomas Jefferson University, Philadelphia, Pennsylvania, 19107, USA

Methods

HFO Detection

HFOs and sharp-spikes were detected in the non-REM sleep iEEG using previously published methods(1–6) implemented in Matlab (Mathworks, Natick, MA, USA). In brief, the HFO detector reduced muscle and electrode artifacts in the iEEG recordings using an independent component analysis (ICA)-based algorithm applied to the referential montage. After applying this ICA-based method, ripples and fast ripples were detected in the referential and bipolar montage recordings per contact by using a Hilbert detector, in which a 1,000th order symmetric finite impulse response (FIR) band-pass filter in the (80–600 Hz) band for ripples and (250-600 Hz) band for fast ripples was applied, and (ii) a Hilbert transform was applied to calculate the instantaneous amplitude of this time series according to the analytic signal z(t)

$$z(t)=a(t)e^{i\phi(t)} \quad (1)$$

where a(t) is the instantaneous amplitude and $\phi(t)$ is the instantaneous phase of z(t). Following the Hilbert transform, the instantaneous HFO amplitude function [a(t)] was smoothed using moving window averaging, the smoothed instantaneous HFO amplitude function was normalized using the mean and standard deviation of the time series, and a statistical threshold defined by the skewness of the normalized time series was used to detect the onset and offset of discrete/potential events.

Selection of a referential or bipolar montage for individual channels or channel pairs, within patients, was based on either visual inspection or automatically selected by the detector. In the former case, patients recorded with Nihon Kohden only have grounding to the first 64 channels and thobe beginning 65 and higher are not, and thus a bipolar montage best reduces noise.In the latter case, machine learning based on several statistical features of the signal were used to automatically transition referential channels to bipolar pairs (http://github.com/shenweiss/).

HFO-like events can arise due to Gibb's phenomenon, i.e., high-pass filtering sharp transients, including epileptiform spikes(7). To distinguish authentic HFOs from authentic HFOs on EEG spikes or spurious HFO due to filter ringing, we used a algorithm that performed topographic analysis of time-frequency plots for each HFO and defined open- and closed-loop contour groups (Figure 1B). The algorithm also measured the power, spectral content, duration, onset time, and offset time of each HFO and categorized the HFO as an HFO on oscillation, HFO on spike, or sharp-spike (i.e. false HFO)(1). HFO on oscillation refers to all HFOs that do not coincide with a spike. Although the Hilbert detector identified putative ripple events using the instantaneous amplitude of the (80-600 Hz) band pass signal, individual definitive ripple events were classified and characterized using the topographic analysis of the time-frequency plots between (80-200 Hz). Coinciding fast ripple and ripple events were characterized by the intersection of the individual events onset and offset time.

Graph Theoretical Measures

All graph theoretical measures were calculated using the Brain Connectivity Toolbox (https://sites.google.com/site/bctnet/)(8,9). For the fast ripple (FR) rate-distance networks, the Euclidian distance was calculated between every electrode contact (i.e. node) using the normalized MNI coordinates. For the resected FR rate-distance networks, the distance between non-resected:resected and non-resected:non-resected nodes were assigned infinite values. The distance between any two nodes that did not both generate a single fast ripple was assigned infinite values. For the remaining nodes the Euclidian distance between the nodes was multiplied by the average rate (events/min) of the events recorded by the two respective nodes. The radius of the network prior to resection and the radius of the resected nework were calculated by deriving the minimum eccentricity across all the connected nodes using the charpath.m function. To calculate the FR rate-distance radius difference the radius of the resected rate-distance network was subtracted from the radius of the whole FR rate-distance network, , prior to resection.

To construct the FR mutual information (MI) networks, event "spike trains" were defined using the onset times of each FR event. Edges were assigned weights using the mutual information between the event spike trains of paired nodes. MI was calculated with the adaptive partition using inter-spike intervals MI estimator (AIMIE) (10), resulting in weighted directed networks. If the MI was zero or not numeric, then the edge was assigned a weight of zero and an infinite distance. For the unresected FR MI networks edges between resected:unresected and resected:resected nodes were assigned a weight of zero and an infinite distance, and for the resected FR MI networks edges between resected:unresected and unresected:unresected nodes were assigned a weight of zero and an infinite distance. To calculate the characteristic path length of the MI network, we inverted the mutual information values to distance and used the charpath.m function.

The characteristic path length ratio was calculated as the path length of the resected FR MI network divided by the path length of the whole network. The clustering coefficient was calculated from the whole FR MI networks using clustering_coef_wd.m. From this calculation the maximum clustering coefficient value among the unresected nodes was evaluated. The local efficiency was calculated from the whole FR MI network, prior to resection, using the updated_efficiency_wei.m function and the mean local efficiency among the unresected nodes was evaluated. The local efficiency was also calculated from the unresected FR MI network, after resection, using the updated_efficiency_wei.m function and the mean value was evaluated. All mean values were calculated by ignoring undefined (i.e. NaN) values and zero values since presumably these were derived from nodes with a FR rate of zero. For the ROC calculations patients with NaN mean or maximum value were ignored. Most of these patients had uncharecterized FR MI networks.

To examine the relationship between FR rate and FR local efficiency we used k-means clustering in Matlab (kmeans.m) using the cityblock distance function and an assumption of three clusters. Within each patient the total number of nodes was calculated for following four node types: 1) the node had a FR rate but had no local efficiency; 2) belonged to cluster 1; 3) belonged to cluster 2; or 4) belonged to cluster 3. We then calculated within patients, and for resected and unresected nodes, the proportion of each type relative to the total number of nodes. A comparison of these proportions was made across the patients stratified by outcome using the Spearman correlation coefficient.

For the five patients in which FR MI networks could not be completely constructed, values were imputed for use in confusion matrices. The Engel 1 patient without a network was imputed as a true positive for graph theoreticlal measures classifying seizure-free outcome. The Engel 2 patient was imputed as a false positive for seizure-free outcome and a true positive for seizure-improved outcome. The three Engel 4 patients were imputed as false positives for both outcomes.

For the control experiment using unweighted networks, the FR MI networks were defined as stated above but edges with a FR MI that was not zero or not numeric were assigned a edge weight and distance of 1 instead of the MI value. The graph theoretical measures described above were used to characterize these networks but the efficiency_bin.m, edge_betweenness_bin.m, clustering_coef_bu.m were used instead.

Fast ripple propagation

For each within-subject pair of electrode contacts generating fast ripples we measured the mutual information (MI)(10) of the fast ripple onset times between the two pairs. If the mutual information was greater than zero, we used the sign test for zero median in Matlab (sign-test.m) to assess unidirectional propagation(11). The MI was measured first to limit the number of potential comparisons. To perform the sign test, we subtracted one contact's FR onset times from the other contact's FR onset times with the onset times of fast ripples in the other contact using the meshgrid.m function.

Differences exceeding ±250 ms were excluded. To limit false detections of propagations due to the high number of channel pairs examined, we used the false discovery rate of 0.05, and a p-value cutoff of 0.005 for the sign test was selected based on multiple comparison testing using the Benjamini-Hochberg false detection ratio (bh_fdr.m) for contact pairs from our dataset with over 120 fast ripples each. This criterion was used to limit the number of comparisons defining the p-value cutoff. As some contacts had less than 120 fast ripples all the edges showing significant propagation were visually inspected, and if fast ripple propagation was not observed they were excluded.

References

1. Waldman ZJ, Shimamoto S, Song I, Orosz I, Bragin A, Fried I, et al. A method for the topographical identification and quantification of high frequency oscillations in intracranial electroencephalography recordings. Clin Neurophysiol. 2018; 129(1):308–18.
2. Weiss SA, Staba RJ, Sharan A, Wu C, Rubinstein D, Das S, et al. Accuracy of high-frequency oscillations recorded intraoperatively for classification of epileptogenic regions. Sci Rep-uk. 2021; 11(1):21388.
3. Weiss SA, Pastore T, Orosz I, Rubinstein D, Gorniak R, Waldman Z, et al. Graph theoretical measures of fast ripples support the epileptic network hypothesis. Brain Commun. 2022; .
4. Weiss SA, Song I, Leng M, Pastore T, Slezak D, Waldman Z, et al. Ripples Have Distinct Spectral Properties and Phase-Amplitude Coupling With Slow Waves, but Indistinct Unit Firing, in Human Epileptogenic Hippocampus. Front Neurol. 2020; 11:174.
5. Shimamoto S, Waldman ZJ, Orosz I, Song I, Bragin A, Fried I, et al. Utilization of independent component analysis for accurate pathological ripple detection in intracranial EEG recordings recorded extra- and intra-operatively. Clin Neurophysiol. 2018; 129(1):296–307.
6. Weiss SA, Berry B, Chervoneva I, Waldman Z, Guba J, Bower M, et al. Visually validated semi-automatic high-frequency oscillation detection aides the delineation of epileptogenic regions during intra-operative electrocorticography. Clin Neurophysiol. 2018; 129(10):2089–98.
7. Bénar CG, Chauvière L, Bartolomei F, Wendling F. Pitfalls of high-pass filtering for detecting epileptic oscillations: A technical note on "false" ripples. Clin Neurophysiol. 2010; 121(3):301–10.
8. Bullmore E, Sporns O. Complex brain networks: graph theoretical analysis of structural and functional systems. Nat Rev Neurosci. 2009; 10(3):186–98.
9. Rubinov M, Sporns O. Complex network measures of brain connectivity: Uses and interpretations. Neuroimage. 2010; 52(3):1059–69.
10. Gribkova ED, Ibrahim BA, Llano DA. A novel mutual information estimator to measure spike train correlations in a model thalamocortical network. J Neurophysiol. 2018; 120(6):2730–44.
11. Otárula KAG, Ellenrieder N von, Cuello-Oderiz C, Dubeau F, Gotman J. High-Frequency Oscillation Networks and Surgical Outcome in Adult Focal Epilepsy. Ann Neurol. 2019; 85(4):485–94.

FR > 350 Hz Rate Percentile

	Engel 1	Engel>1
FR Resected	4(1)	4(2)
FR intact	6(9)	9(11)

	Engel <4	Engel=4
FR Resected	7(2)	1(1)
FR intact	7(12)	8(8)

All FR Rate

	6(1)	4(2)
	4(9)	9(11)

	7(2)	3(1)
	7(12)	6(8)

FR+R Rate

	4(2)	6(5)
	6(8)	7(8)

	7(4)	3(3)
	7(10)	6(6)

Figure S1: Confusion matrices of seizure-free classification (left) and seizure-improved classification (right) based on complete resection of contacts with FR rates in the 99th percentile of the FR rate distribution, or 97.5th percentile (in parenthesis), using FR>350 Hz (top), all FR (middle), or FR superimposed on ripple(R) rates.

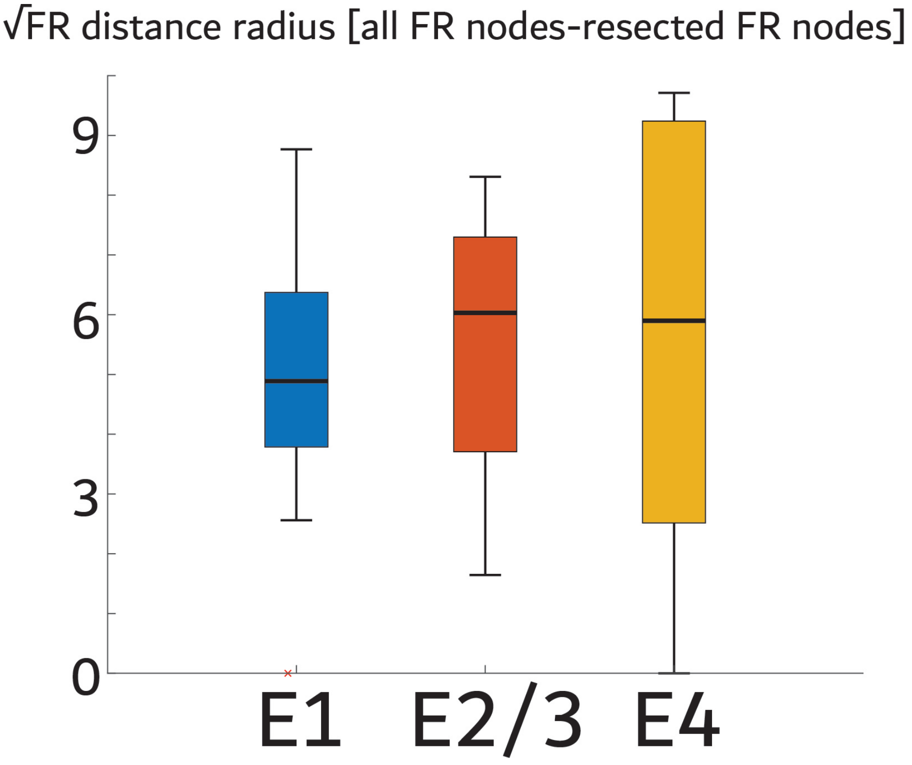

Figure S2: The unweighted fast ripple (FR) radius difference of the distance network formed by all FR nodes subtracted from the distance network of the resected FR nodes does not correlate with outcome. The unweighted FR distance radius difference was calculated as in Figure 3, but the edges were not weighted by the mean FR rates between the respective nodes. The square root of the radius difference, stratified by outcome for all the patients in the cohort, is shown.

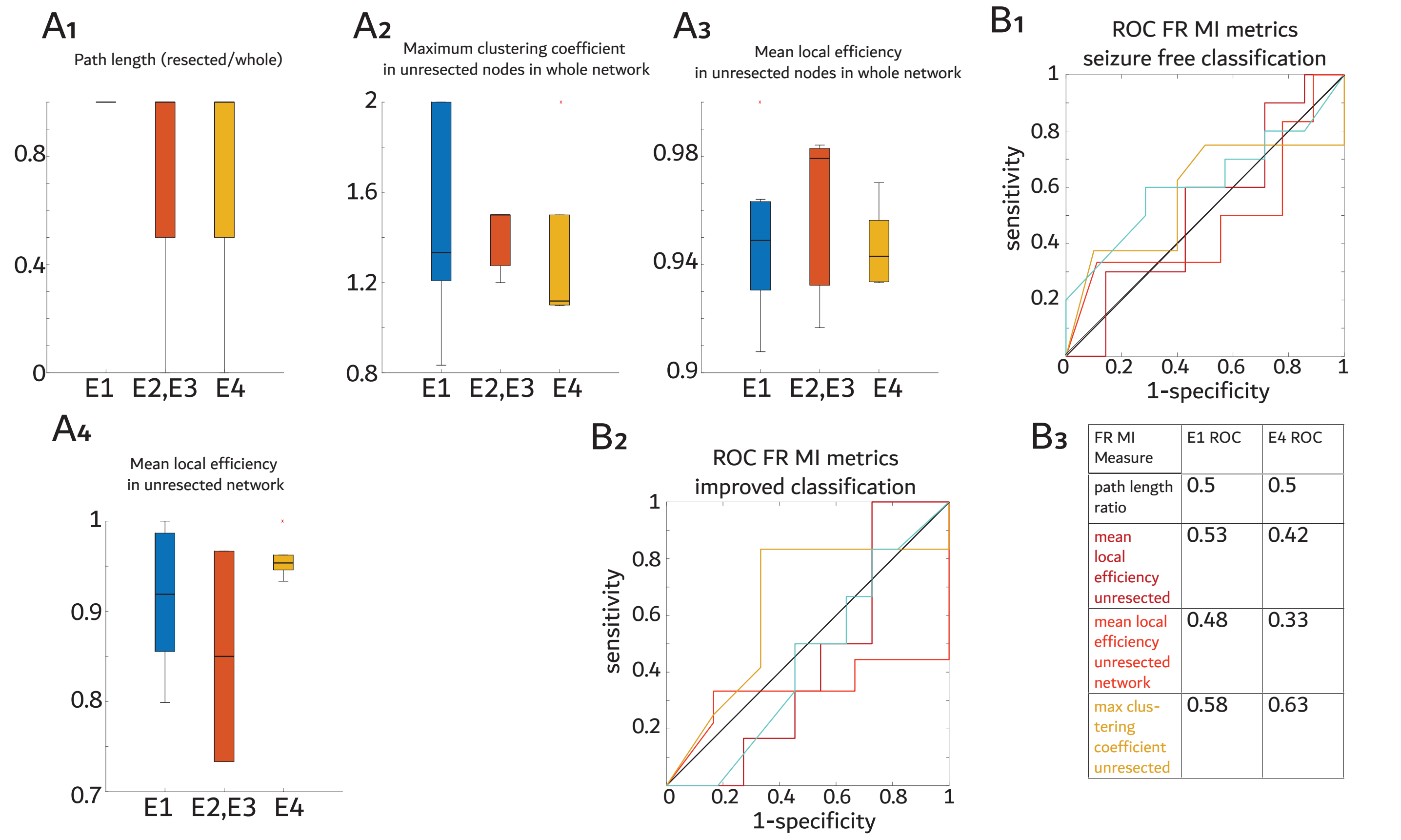

FR MI Measure	E1 ROC	E4 ROC
path length ratio	0.5	0.5
mean local efficiency unresected	0.53	0.42
mean local efficiency unresected network	0.48	0.33
max clus-tering coefficient unresected	0.58	0.63

Figure S3: Control experiment demonstrating that graph theoretical metrics of the fast ripple (FR) **unweighted** correlational network constructed from the edges and respective nodes with FR mutual information>0 (Figure 5-7) does not predict post-operative seizure outcome. (A) Box plots of the FR unweighted graph theoretical metrics stratified by post operative seizure outcome Engel (E) class. The FR unweighted characteristic path length resection ratio (A1) measured from known communicating edges only in the resection margins divided by the path length measured from the whole FR unweighted network. The maximum clustering coefficient value (A2) among the unresected nodes calculated from the whole FR unweighted network prior to resection. The mean local efficiency value (A3) as in A2. The mean local efficiency value from the FR unweighted network characterized after resection (A4). (B) Receiver operating characteristic curves (ROC) of these FR unweighted graph theoretical metrics for preding seizure free (E1) outcome (B1) or improved outcome (B2). The color coded legend for the ROC curves and the corresponding area under the ROC curve values (B3). Note two patients were excluded because no FR MI network could be characterized, and another three patients, who were not seizure-free, were excluded because the entire FR MI network was resected.

Table S1: Patient characteristics in the study cohort. Abbreviations M: male, F: female, L: left, R: right, , N/A: not applicable, ATL: anterior temporal lobectomy, MT(L): mesial temporal lobe, MTS: mesial temporal sclerosis, SMA: supplementary motor area, TBI: traumatic brain injury, LOC: loss of consciousness, RNS: responsive neurostimulator, VNS: vagal nerve stimulator, SUDEP: sudden unexpected death in epilepsy, @: time to last follow up.

ID / age / sex	Risk Factor	MRI	PET (hypometabolic)	iEEG clinical consensus SOZs	Surgery	Path.	Outcome
IO01 55/F	minor TBI	Normal	L temporal	L MT	modified L ATL hippocampus sparing	Gliosis	Engel IA@24 months
IO08 22/F	hypertensive encephalopathy	post L ATL	N/A	L middle temporal gyrus	modified L temporal lobectomy (posterior temporal included)	Gliosis	Engel 1A @48 months
IO18 28/M	Minor TBI	Normal	Normal	Right insula, cuneus, inferior and middle frontal gyrus	R. Frontal lobe	Gliosis	Engel IA@24 months
4122 19/M	None	Normal	R temporal	R Inferior temporal gyrus	modified R temporal lobectomy (posterior temporal included)	Gliosis	Engel IA@24 months
4124 18/F	None	L MTL whitematter hyper-intensity	Normal	R SMA	R frontal lobe resection	cortical dysplasia	Engel IA@24 months
4145 25/M	None	Normal	Normal	L cingulate gyrus, medial frontal gyrus, middle frontal gyrus, superior frontal gyrus	L frontal lobectomy	cortical dysplasia	Engel IA@40 months
4166 49/F	meningitis	Encephalo-malacia	L temporal	L MT, uncus, superior temporal gyrus, frontal lesion	L temporal and frontal lobe resection	gliosis	Engel IB@42 months
IO12 31/F	none	1 cm pineal cyst	R lateral temporal	L MT	Modified L temporal lobectomy (posterior temporal included)	Gliosis	Engel IIB@24 months

453 47/M	None	T2 hyper-intensity in R temporal pole > L frontal pole. Inferior portion of R temporal pole with blurred gray-white matter border	R temporal	R MT	R anterior ATL	Cortical dysplasia IIb	Engel IA@60 months
456 37/F	None	Normal	R temporal	Bilateral MT, middle temporal gyrus R>L	modified R ATL (preserved middle and superior temporal gyrus)	gliosis	Engel IVC@48 months
462 26/M	TBI, family history	left superior temporal gyrus encephalomalicia	L parieto-occipital	L temporal neocortical, L frontal	modified LATL hippocampus sparing	gliosis	Engel IV@6 months RNS placed and revised
466 21/F	None	Normal	L temporal	R fusiform gyrus, superior temporal gyrus, uncus	R ATL	MTS	Engel IB@35 months
473 70/F	TBI w/ LOC	L MTS, extra-temporal T2	L temporal and frontal	L MT, fusiform gyrus, uncus	L MT visualase	N/A	Engel IIIA@18 months
477 45/F	None	periventricular nodular heterotopia, right frontal T2	R temporal	R MT	ATL	gliosis	Engel IB@31 months
479 36/M	TBI w/ LOC	Encephalomalacia	R temporal	R insula, bilateral middle temporal gyrus, superior temporal gyrus	modified R ATL (posterior temporal and temporal-parietal-occipital junction included.	gliosis	Engel IVB@33 months

IO21 50/F	None	Prior R. ATL	N/A	Right orbitofrontal cortex.	R. Frontal lobe	hippocampal sclerosis, cortical dysplasia	Engel IVB@24 months
4110 42/F	encephalitis	Encephalomalacia	Normal	L inferior frontal gyrus, insula, MT	L temporal lobe and insula resection	Gliosis	SUDEP @6 weeks
IO23 40/M	Significant head injury with LOC	Left temporal T2 hyperintensity with mild enhancement	N/A	Bilateral MT, right lateral temporal	L temporal lobectomy, anterior thalamic DBS	Gliosis	Engel IVB@24 months
IO13 40/M	None	R parietal lobe resection	R parietal and R occipital	R insula, precuneus, middle occipital gyrus, superior parietal lobule, superior occipital gyrus, superior temporal gyrus, middle temporal gyrus	R parietal	gliosis	Engel IIIA@18 months
IO15 37/M	None	L posterior fossa arachnoid cyst, R ATL	R temporal	L MT, R cingulate, post. cingulate, mesial frontal, precuneus	R anterior cingulate thermal ablation	gliosis	Engel IVB@36 months
IO19 39/M	None	Prior R parietal resection	R parietal and occipital hypometabolism	R parietal lobe	R. Parietal lobe resection	Gliosis	Engel IVB@36 months

Table S2: Patient iEEG implant, fast ripple (FR) contact, and spatial extent of the resection and SOZ characteristics in the study cohort. A FR contact is defined as a contact with at least one FR event during the recording duration. The resection radius and SOZ radius was determined from the normalized MNI coordinates of the resected and SOZ electrodes. Resected territory unsampled by electrode contacts are not included in these measures.

ID	implant unilateral/ bilateral	total # contacts	>350 Hz FR contacts (unresected)	resection radius in mm	SOZ radius (unresected) in mm	Outcome
IO01	bilateral	154	11(4)	95.8	19.6 (19.6)	Engel IA@24 months
IO08	bilateral	110	15(10)	98.1	42.7 (0.0)	Engel 1A @48 months
IO18	bilateral	155	17(7)	81.2	58.6 (31.6)	Engel IA@24 months
4122	unilateral	149	9(7)	67.7	45.6 (45.6)	Engel IA@24 months
4124	bilateral	114	5(4)	60.5	2.6(2.6)	Engel IA@24 months
4145	bilateral	113	42(25)	67.8	53.3 (30.9)	Engel IA@40 months
4166	unilateral	124	9(7)	60.1	22.2 (0.0)	Engel IB@42 months
IO12	bilateral	166	6(6)	93.1	16.1 (2.1)	Engel IIB@24 months
IO05	unilateral	154	61(47)	72.4	53.3 (36.0)	Engel IVB@40 months

453	bilateral	65	14(10)	90.6	27.0 (1.1)	Engel IA@60 months
456	bilateral	73	8(5)	96.7	12.4 (0.0)	Engel IVC@48 months
462	bilateral	59	14(12)	82.0	20.0 (4.2)	Engel IV@6 months RNS placed and revised
466	bilateral	62	11(7)	89.6	43.4 (31.1)	Engel IB@35 months
473	bilateral	59	4(2)	84.2	7.7 (0.0)	Engel IIIA@18 months
477	bilateral	50	25(21)	87.3	10.4 (0.0)	Engel IB@31 months
479	bilateral	48	9(8)	90.7	114.8(104.2)	Engel IVB@33 months
469	bilateral	48	19(17)	83.5	51.4 (38.7)	Engel IIIA@63 months

IO21 50/F	unilateral	109	6(3)	61.7	31.0 (0.0)	Engel IVB@24 months
4110 42/F	unilateral	147	13(4)	54.7	41.0 (25.1)	SUDEP @6 weeks
IO23	bilateral	136	36(36)	94.0	52.6 (52.6)	Engel IVB@24 months
IO13	unilateral	164	22(17)	72.9	38.8 (29.9)	Engel IIIA@18 months
IO15	bilateral	225	11(11)	90.7	84.5 (80.8)	Engel IVB@36 months
IO19	unilateral	93	28(15)	60.4	59.1 (0.0)	Engel IVB@36 months

Table S3: Generalized linear mixed effects models results of fast ripple (FR) on oscillation frequency using the electrode contacts resection status and location as predictors. The three models are stratified by post-operative seizure outcome status (Engel). For Engel 1 (seizure free patients) resected contacts predicted a higher FR frequency, For Engel >1 (non-seizure free) patients resected contacts predicted a lower FR frequency.

Fast Ripple on Oscillation Frequency (Hz)	Intercept Estimate	Intercept p-value	Resection Estimate	Resection p-value	Location Estimate	Location p-value
Engel 1 patients (N=10, n=13,299)	5.502 [5.41 5.6]	<1e-999	0.096 [0.09 0.10]	<1e-163	0.016 [0.15 0.02]	<1e-98
Engel 2/3 patients (N=4, n=3,605)	5.587 [5.51 5.67]	<1e-999	-0.050 [-0.07 -0.03]	<1e-8	-0.001 [-0.006 [0.003]	n.s.
Engel 4 patients (N=9, n=24,329)	5.680 [5.57 5.79]	<1e-999	-0.300 [-0.31 -0.29]	<1e-999	0.001 [e3-4 2e-3]	<0.01

Table S4: Revised classification, using the graph theoretical FR resection measures, of patients misclassified as seizure-free (top) and improved (bottom) by the fast ripple (FR >350 Hz) resection ratio (RR) at Youden's J. Plus (+) indicates a correct revised classification of non-seizure free (top) or non-responder (bottom) by the graph theoretical FR measure at Youden's J, Minus (-) indicates an incorrect revised classification. Not applicable (n/a) refers to patients without a FR MI network, or a completely resected FR MI network and an indeterminate test result.

**Non-seizure free patients**	**FR rate-distance**	**FR MI Path length**	**FR MI Clust. Coeff.**	**FR MI Local Eff.**	**FR MI Local Eff. UR.**
IO012	+	-	+	+	+
469	+	+	+	+	+
473	-	n/a	n/a	n/a	n/a
IO005	+	+	+	+	+
IO021	-	n/a	n/a	n/a	n/a
456	-	n/a	n/a	n/a	n/a
IO013	-	-	+	+	-
IO019	+	+	+	+	+
**Non-responder Patients**	**FR rate-distance**	**FR MI Path length**	**FR MI Clust. Coeff.**	**FR MI Local Eff.**	**FR MI Local Eff. UR.**
IO005	+	+	+	+	+
IO021	-	n/a	n/a	n/a	n/a
456	-	n/a	n/a	n/a	n/a
IO019	+	+	-	+	+
